# A critical role of hepatitis B virus polymerase in cirrhosis, hepatocellular carcinoma, and steatosis

**DOI:** 10.1002/2211-5463.12357

**Published:** 2017-12-19

**Authors:** Hea‐Jong Chung, Xiao Chen, Yang Yu, Heui‐Kwan Lee, Chang Ho Song, Han Choe, Seungkoo Lee, Hyeon‐Jin Kim, Seong‐Tshool Hong

**Affiliations:** ^1^ Department of Biomedical Sciences Chonbuk National University Medical School Jeonju Chonbuk South Korea; ^2^ Department of Radiation Oncology Presbyterian Medical Center Seonam University Medical School Jeonju Chonbuk South Korea; ^3^ Department of Anatomy Chonbuk National University Medical School Jeonju Chonbuk South Korea; ^4^ Department of Physiology Bio‐Medical Institute of Technology Asan Medical Center University of Ulsan College of Medicine Seoul South Korea; ^5^ Department of Anatomic Pathology School of Medicine Kangwon National University Chuncheon Gangwon South Korea; ^6^ JINIS BDRD Institute JINIS Biopharmaceuticals Co. Wanju Chonbuk South Korea; ^7^ Present address: Department of Microbiology Seonam University Medical School Namwon Chonbuk South Korea

**Keywords:** chronic hepatitis B virus, cirrhosis, hepatocellular carcinoma, reverse transcriptase, steatosis

## Abstract

Hepatitis B is one of the most common infectious diseases in the world; more than 350 million people are carriers of hepatitis B virus (HBV). Chronic HBV infection (CHB) leads to liver diseases such as cirrhosis, hepatocellular carcinoma (HCC), and steatosis. Despite its seriousness in terms of public health, the pathogenic mechanism of how CHB leads to liver diseases, especially cirrhosis and steatosis, remains unclear. We studied the role of HBV polymerase (HBp) reverse transcriptase (RT) activity in association with the pathogenesis of liver diseases in CHB by developing transgenic mice expressing HBp or the RT domain of HBp. Thorough pathological, serological, and histological analyses of the transgenic mice, as well as mechanistic studies, were conducted. All of the transgenic mice expressing RT in their livers developed early cirrhosis with steatosis by 18 months of age, and 10% developed HCC. The RT activity of HBp stimulates coordinated proapoptotic and proinflammatory responses involving the caspase‐9, caspase‐3, and caspase‐1 pathways that might lead to the development of cirrhosis, HCC, and steatosis. The animal model described here should prove useful for elucidating the molecular events in the CHB‐induced liver diseases.

**Abbreviations**
AHR: aryl hydrocarbon receptor
ALB: blood levels of albumin
ALT: alanine aminotransferase
AST: aspartate aminotransferase
CHB: chronic hepatitis B virus infection
CREB: cyclic AMP‐responsive element‐binding protein
H&E: hematoxylin and eosin
HBc: HBV core
HBp: HBV polymerase
HBsAg: HBV surface antigen
HBs: HBV surface
HBV: hepatitis B virus
HBx: HBV transcriptional regulatory protein
HCC: hepatocellular carcinoma
ICAM‐1: intercellular adhesion molecule‐1
In: inflammation
LFT: liver function test
LV: lipid vacuoles
MB: Mallory bodies
MCP‐1: monocyte chemotactic protein‐1
MT: Masson trichrome
RBP5: retinol‐binding protein 5
RT: reverse transcriptase
TBP: TATA‐binding protein
TGF‐β1: transforming growth factor‐β1
TIMP‐1: tissue inhibitor of metalloproteinase‐1
TUNEL: terminal deoxynucleotidyl transferase‐mediated dUTP‐biotin nick‐end labeling

Hepatitis B virus (HBV) is the most frequent pathogen that infects humans and chimpanzees, causing acute and chronic hepatitis or liver inflammation 1. After initial infection with HBV, most patients successfully develop immunity to clear the infection (87–90%), but the rest become chronic HBV carriers 2. Patients who are infected with HBV as neonates or young children usually become chronically infected 2. Chronic hepatitis B virus (CHB) infection progresses nonlinearly through three to four phases: the immune‐tolerant phase, the immune clearance or immunoactive phase, the nonreplicative inactive phase, and the possible reactivation phase 2, 3.

The public health consequences of CHB are very serious. More than 350 million people worldwide have CHB, and 600 000 deaths are estimated to occur annually as a result of CHB‐induced liver diseases, such as hepatocellular carcinoma (HCC) or cirrhosis 3. Host‐mediated inflammation in the liver plays a direct role in the pathogenesis of acute HBV infection. However, unlike acute HBV infection, host‐mediated inflammation alone cannot explain the pathogenic consequences of CHB, such as HCC, cirrhosis, or steatosis. In a typical CHB case, HBV is not recognized as a foreign material by the host immune system to cause direct immunological reactions in the liver 4. Therefore, CHB itself is not cytotoxic and causes no symptoms. Because HBV is not recognized by the host immune system in CHB, CHB carriers typically have very high HBV levels in their bodies 5.

Because the host‐mediated inflammation that explains acute HBV infection does not explain the pathogenesis of CHB‐induced liver diseases, such as HCC, cirrhosis, or steatosis, pathogenic factors encoded in the HBV genome have been actively investigated to elucidate how CHB causes liver diseases. HBV contains a small partially double‐stranded circular DNA. This DNA contains only four overlapping open reading frames, which encode the surface (HBs), core (HBc), polymerase (HBp), and transcriptional regulatory proteins (HBx) 6. Among these four genes, HBx has been most actively investigated as an etiological agent of CHB‐induced liver diseases. Transgenic mouse experiments have provided insight into the potential role of HBx in CHB‐induced liver diseases, but the results obtained so far have been inconsistent. HBx was shown to promote HCC formation in some transgenic mice 7. However, most experiments showed that transgenic mice expressing HBx developed HCC only after treatment with carcinogens rather than HCC directly induced by HBx, indicating that HBx was only able to sensitize the host hepatocytes to carcinogens 8, 9, 10. Based on these experimental results, it would be reasonable to conclude that HBx mostly acts as a cofactor in carcinogen‐induced hepatocarcinogenesis 8, 11.

Hepatitis B virus polymerase is a broad‐range transactivator that can stimulate the transcription of its own HBV genes as well as various host genes, including proto‐oncogenes, genes related to intracellular signal transduction pathways, genes regulating the cell cycle, and genes related to the apoptotic signaling pathway 11. In addition to the ability of HBx to bind various heterologous promoters in hepatocytes and stimulate gene transcription, HBx can also directly bind various host proteins, including cyclic AMP‐responsive element‐binding protein (CREB), activating transactivation factor 1, the tryptase TL2, TATA‐binding protein (TBP), retinol‐binding protein 5 (RBP5), components of the proteasome complex, UV‐DDP‐p127, a subunit of the aryl hydrocarbon receptor (AHR) core complex, and a novel inhibitor protein of HBx transactivation 11. Despite the enormous functional diversity of HBx, none of these pathways are directly involved in HCC development; thus, a pathway for cell transformation and HCC development has not yet been identified. More importantly, under no conditions did any HBx‐expressing transgenic mice develop cirrhosis, which is much more prevalent than HCC in CHB. Therefore, the pathogenic mechanism of how CHB leads to liver diseases, especially cirrhosis, remained unknown until now.

Hepatitis B virus is a pararetrovirus that replicates its genome via the reverse transcription of an RNA intermediate, meaning that the reverse transcriptase (RT) embedded in HBp plays an essential role in the life cycle of HBV 12. One common characteristic of retroviruses, pararetroviruses, and retroviral vectors is that RT is encoded in their genomes. At the same time, all chronic infections caused by retroviruses, pararetroviruses, such as HBV, and even retroviral vectors designed for gene therapy eventually become deleterious to the host 13, 14. As all retroviruses, pararetroviruses, and retroviral vectors encode RT, in this study, we investigated the possible role of HBp RT activity in association with the pathogenesis of liver diseases in CHB by creating transgenic mice. Our experiments showed that HBp RT is a key etiological agent for CHB‐induced liver diseases.

## Materials and methods

### Cloning of HBp and RT constructs for liver‐specific expression in transgenic mice

The DNA segments for *HBp* and *RT* were amplified by PCR from the genomic DNA extracted from the tumor tissue of an HBV surface antigen (HBsAg) carrier 15, 16 with HBp‐F, HBp‐R, RT‐F, and RT‐R primers (Table S1). The PCR‐amplified *HBp* and *RT* were 2.5 kb and 1.2 kb, respectively, and these genes were cloned into pcDNA3.1 (+) vectors. To insert a liver‐specific promoter upstream of *HBp* or *RT*, the 1.3‐kb DNA segment of the alpha‐1‐anti‐trypsin gene promoter (GenBank accession No. D38257; AAP) was first amplified by PCR from genomic DNA extracted from a C57BL/6N mouse with AAP‐F and AAP‐R primers (Table S1). Then, the DNA fragment of AAP was ligated upstream of the *HBp* or *RT* gene in the pcDNA3.1 (+) vectors. The *AAP‐RT* and *AAP‐HBp* fusion plasmids in *Escherichiea coli* DH5α were purified from the *E. coli* culture by NucleoBond Xtra Midi Plus (Macherey‐Nagel, Düren, Germany). The DNA sequences of the *AAP‐RT* and *AAP‐HBp* fusion plasmids were confirmed by DNA sequencing (Genotech, Daejeon, South Korea) before the generation of the transgenic mice.

### Establishment of transgenic mice

After the digestion of the pcDNA3.1(+)‐*AAP‐HBp* or pcDNA3.1(+)‐*AAP‐RT* plasmids with *SnaBI* and *SmaI* restriction enzymes, the 5.3‐kb DNA fragment containing *AAP‐HBp* or the 3.9‐kb DNA fragment containing *AAP‐RT* was separated by agarose gel electrophoresis. The 5.3‐kb DNA fragment containing *AAP‐HBp* or the 3.9‐kb DNA fragment containing *AAP‐RT* was purified using an Expin™ CleanUp kit (GeneAll, Seoul, South Korea), and each of these DNA fragments was microinjected into the pronuclei of fertilized mouse eggs. The embryos were implanted in the uterus of a surrogate mother with a C57BL/6N genetic background. The transgenic mice were backcrossed with the C57BL/6N strain, and the offspring were used in this study. The animal studies were conducted in an age‐matched manner for all experiments. All efforts were made to minimize suffering. All animals were maintained under a 12‐h light/dark cycle throughout the experiments. The environment was maintained at a temperature of 22 ± 1 °C and a humidity of 40–50%.

All animal care and use protocols were performed strictly in accordance with the ethical guidelines of the Ethics Committee of the Chonbuk National University Laboratory Animal Center, and the animal study protocol was approved by the institution (Approved Number: CBU 2012‐0040) in accordance with the ‘Guide for the Care and Use of Laboratory Animals’, published by the National Research Council and endorsed by the ARRIVE Guidelines.

### Genotyping of the transgenic mice

Mouse tails were digested in TE buffer containing proteinase K (Macherey‐Nagel) overnight at 56 °C. After the addition of chaotropic salts and ethanol to the digested samples, genomic DNA bound to the columns provided in the kit. Contaminants were removed by subsequent washing with two different buffers, and pure genomic DNA was eluted under low ionic strength conditions in elution buffer (Macherey‐Nagel). PCR was performed using a Bio‐Rad C1000 Thermal Cycler (Bio‐Rad, Hercules, CA, USA) under the following conditions: initial denaturation at 94 °C for 5 min; DNA amplification at 94 °C for 20 s, 49 °C for 30 s, and 72 °C for 2 min for 35 cycles; and final extension for 5 min at 72 °C. The PCR primers used were HBp1‐F, HBp1‐R, RT1‐F, and RT1‐R (Table S1).

### Gene expression analysis of the transgenic mice

Total RNA was isolated from transgenic livers stored in RNAlater (Life Technologies, Carlsbad, CA, USA). cDNA was synthesized using a Bio‐Rad C1000 Thermal Cycler using a PrimeScript™ RT Reagent kit (Takara, Shiga, Japan) under the following conditions: 37 °C for 15 min and 85 °C for 5 s. The samples were then stored at 4 °C until they were used in additional experiments. Real‐time PCR was performed using an Applied Biosystems 7900HT Fast Real‐Time PCR instrument in accordance with the manufacturer's protocol with a SYBR Premix Ex Taq™ kit (Takara). The thermal cycling program was 94 °C for 5 min; 94 °C for 20 s, 49 °C for 30 s, and 72 °C for 2 min for 35 cycles; and a final extension for 5 min at 72 °C. The primers used for real‐time PCR were HBp2‐F, HBp2‐R, RT2‐F, RT2‐R, 18S rRNA‐F, and 18S rRNA‐R (Table S1). The primers were designed using primer express 3.0 software (ThermoFisher Scientific, Watham, MA, USA), and data were analyzed using rq manager 1.2 software (Applied Biosystems, FosterCity, CA, USA) according to the ΔΔCt method. Amplified cDNA derived from 18S rRNA was used as an endogenous control.

### Genomic localization of the transgenes

The integration loci and copy numbers were determined by alternative PCR utilizing genomic DNA isolated from mouse tails. A set of nested specific primers (SP primers) and arbitrary degenerate primers (AD primers) was designed for sequencing the flanking sequences of transgenes. SP primers that could specifically bind the transgene for synthesizing new strands using the transgene as a template harbored high melting temperatures (*T*_m_, 60–65 °C) to achieve exclusively specific amplification. AD primers that were aimed to bind randomly to the mouse genome harbored low *T*_m_ (43–45 °C) to generate many more amplicons with or without specificity. The primary alternative PCR was performed using a Bio‐Rad C1000 Thermal Cycler under the following conditions: initial denaturation at 95 °C for 3 min; 15 cycles of 95 °C for 10 s, *T*_m_ (SP) 60–65 °C for 10 s, 72 °C for 40 s and 95 °C for 10 s; 43–45 °C for 10 s; 72 °C for 40 s; final extension at 72 °C for 3 min; and hold at 4 °C. Without purification, 2 μL of the 10‐fold‐diluted PCR solution from the primary PCR was used as the template for the secondary alternative PCR. After secondary alternative PCR was performed under the same conditions, the final PCR solutions were subjected to gel electrophoresis in 1× TAE buffer (40 mm Tris, 20 mm acetic acid, and 1 mm EDTA, pH 8.3). After electrophoresis, the DNA bands on the agarose gel were purified using an Expin™ CleanUp kit (GeneAll). The purified DNA amplicons were sequenced with the SP primers used for alternative PCR. The SP primers were designed using primer premier 6.0 software (Premier Biosoft International, Palo Alto, CA, USA), and sequence data were analyzed using bioedit Sequence Alignment Editor Software (Informer Technology Inc., Roseau Valley, Dominica) and compared with the *mus musculus* genome using BLAST. The primers used in alternative PCR were SP1‐R, SP2‐R, SP3‐F, SP4‐F, AD1‐r, AD2‐r, and AD3‐r (Table S1).

### Blood profile analysis

All serum enzyme levels were assessed by ELISA kits in accordance with the manufacturer's instructions. The ELISA kits were purchased from Quantikine R&D Systems [monocyte chemotactic protein 1 (MCP‐1), tissue inhibitor of metalloproteinase‐1 (TIMP‐1), transforming growth factor‐β1 (TGF‐β1); Minneapolis, MN, USA], USCNK Life Science [alanine aminotransferase (ALT), aspartate aminotransferase (AST); Huston, TX, USA], and MyBioSource [intercellular adhesion molecule‐1 (ICAM‐1), HA, Col‐IV, blood levels of albumin (ALB); San Diego, CA, USA]. Serum total cholesterol (TC), high‐density lipoprotein (HDL) cholesterol, low‐density lipoprotein (LDL) cholesterol, and triacylglycerol (TG) assays were performed according to the standard protocols (Asanpharm, Seoul, South Korea). Blood glucose was tested by Accu‐Chek 296 (Roche, Basel, Switzerland). The plate was read at a specific wavelength using an iMark™ Microplate reader (Bio‐Rad).

### Histology of liver tissue samples

Liver tissue samples of mice were isolated randomly from the transgenic and control groups and fixed in 10% neutral buffered formalin (Sigma‐Aldrich, St. Louis, MO, USA) after washing with 0.9% normal saline. The specimens were routinely processed, embedded in paraffin wax, cut into 5‐μm‐thick sections, and stained with hematoxylin and eosin (H&E; Sigma‐Aldrich) and Masson trichrome (MT; Sigma‐Aldrich) in accordance with typical protocols. The H&E and MT histological images were obtained using an Aperio Scanscope (Aperio Technologies, Vista, UK) and processed by ImageScope software. Images of the terminal deoxynucleotidyl transferase‐mediated dUTP‐biotin nick‐end labeling (TUNEL) staining were obtained with an Olympus VANOX‐T microscope (Olympus Corporation, Shinjuku, Tokyo, Japan) and Nikon camera (Nikon, Minato, Tokyo, Japan), captured with act‐2u 1.5 software (Nikon), and processed by image‐pro Plus software (Media Cybernetics Inc., Rockville, MD, USA); the images were manipulated for contrast enhancement.

### TUNEL assay

Apoptosis was assayed by TUNEL staining (GenScript, Piscataway, NJ, USA). After the paraffin sections were deparaffinized and rehydrated in a serial ethanol gradient, the slides were immersed in a 0.85% sodium chloride solution for 5 min, followed by a 15‐min fixation in 10% buffered formalin at room temperature. Then, 50 μL of a proteinase K solution (GenScript) was added to digest the samples during 25‐min incubation at room temperature; then, the fixation step was repeated. Subsequently, the slides were incubated with 3% H_2_O_2_ for 10 min at room temperature. Next, 50 μL of freshly prepared TUNEL reaction mixture was added, and the slides were incubated at 37 °C for 60 min with a coverslip in a wet chamber. Subsequently, the slides were incubated with 50 μL of a horseradish peroxidase‐conjugated streptavidin solution for 30 min at 37 °C, stained with 50 μL of freshly prepared 2,4‐diaminobutyric acid for 3 min at room temperature, mounted with Gel/Mount (Biomeda, Foster City, CA, USA), and sealed with nail polish.

### Caspase staining

Freshly isolated hepatocytes were washed with chilled 1× PBS three times and stained with a caspase‐1 probe (ImmunoChemistry Technologies, Bloomington, MN, USA), a caspase‐3 probe (BD Biosciences, San Jose, CA, USA), and a caspase‐9 probe (Abcam, Cambridge, UK) following the manufacturer's instructions. Then, the cells were analyzed by flow cytometry directly or fixed with 1% paraformaldehyde for 2 h, stained with DAPI for 15 min at room temperature, and washed three times with 1× PBS. The samples were then sent for flow cytometry analysis or slide preparation. Twenty microliters of the cell suspension was added to the slides and spun at 845 ***g*** for 5 min. The slides were mounted with Gel/Mount (Biomeda), sealed with nail polish, and stored at 4 °C in the dark.

### Flow cytometry analysis

Samples were stained and examined using flow cytometry and cellquest software (BD Bioscience), and the data were analyzed with winmdi 2.9 software (De Novo Software, Glendale, CA, USA). Flow cytometry quality control, including alignment, calibration, and color compensation, was performed before sample acquisition according to the manufacturer's instructions. Samples with caspase‐9 and caspase‐3 staining or blank vector transfection were prepared for only FL1–FL2 compensation. The cell suspension used for the flow cytometry analysis was diluted to ~ 10^−6^ cells·mL^−1^, and samples were pipetted up and down before testing.

### Confocal microscopy

Confocal images were captured using a confocal laser scanning microscope (Carl Zeiss, Jena, Germany), the LSM 510 META module, and 20× lenses. Caspase‐3 and caspase‐9 fluorescence was excited using a 488‐nm laser and emitted at 570 nm. Caspase‐1 fluorescence was excited using a 633‐nm laser and emitted at 660 nm. All images were equally processed with contrast enhancement. Images were processed using the Start LSM Image Browser.

### Isolation of primary hepatocytes

Hepatocytes were isolated from control and transgenic mice at 6 and 18 months using the two‐step liver perfusion method 17. After the mice were anesthetized with ether, a needle was quickly inserted into the inferior vena cava, and a small incision was made in the portal vein to drain the blood and perfusion medium. Liver perfusions were performed with 50 mL of prewarmed 1× liver perfusion medium (Life Technologies) at a speed of 130 r.p.m. using a peripheral pump (Manostat^®^, Barrington, IL, USA); then, the livers were digested with 50 mL of prewarmed 1× liver digestion medium (Life Technologies) at the same speed. After the digestion was completed, as indicated by light yellow and much softer than normal liver surfaces, the livers were excised, placed in a culture dish, and washed with 1× PBS. The isolated livers were then immersed in chilled hepatocyte wash medium (Life Technologies) and transferred to a clean bench for further experiments. The membranes of the digested livers were gently removed using forceps, and the liver lobes were then shaken slowly to release free hepatocytes. The hepatocyte suspensions were filtered with a 100‐μL cell strainer (SPL LifeSciences, Seoul, South Korea) to remove small pieces of tissue; then, the hepatocytes were purified through gradient centrifugation at speeds of 200, 100, and 50 ***g*** for 3 min. The purified hepatocytes were resuspended in 10 mL of wash medium before further experiments.

### RT assay

The hepatocytes isolated by the liver perfusion method were suspended in cell lysis buffer [20 mm Tris/HCl pH 8.0 solution containing 150 mm sodium chloride, 1 mm EDTA, 1% Triton X, 1% NP‐40, 1 mm AEBSF, and 1 protease inhibitor cocktail tablet (Roche)] and ultrasonicated (Saehan Ultrasonic, Seoul, South Korea) for 10 s 5 times with 1‐min intervals. After centrifuging the cell lysis solutions at 13 523 ***g*** for 20 min to remove cell debris, the supernatants were transferred to fresh tubes for the RT assay. The RT assays were performed according to the instructions of the EnzChek^®^ RT Assay kit (E‐22064; Molecular Probes, Eugene, OR, USA). The fluorescence signals of the reactions were detected using a microplate reader (Bio‐Rad).

### Statistical analysis

Statistical significance was determined using spss 18.0 (IBM Corporation, Armonk, NY, USA). The data are presented as the mean ± SEM and were compared using paired Student's *t*‐tests. *P* values ≤ 0.05 were considered significant.

## Results

### Establishing transgenic mice expressing HBp or the HBp RT domain specifically in the liver

**Figure 1:**
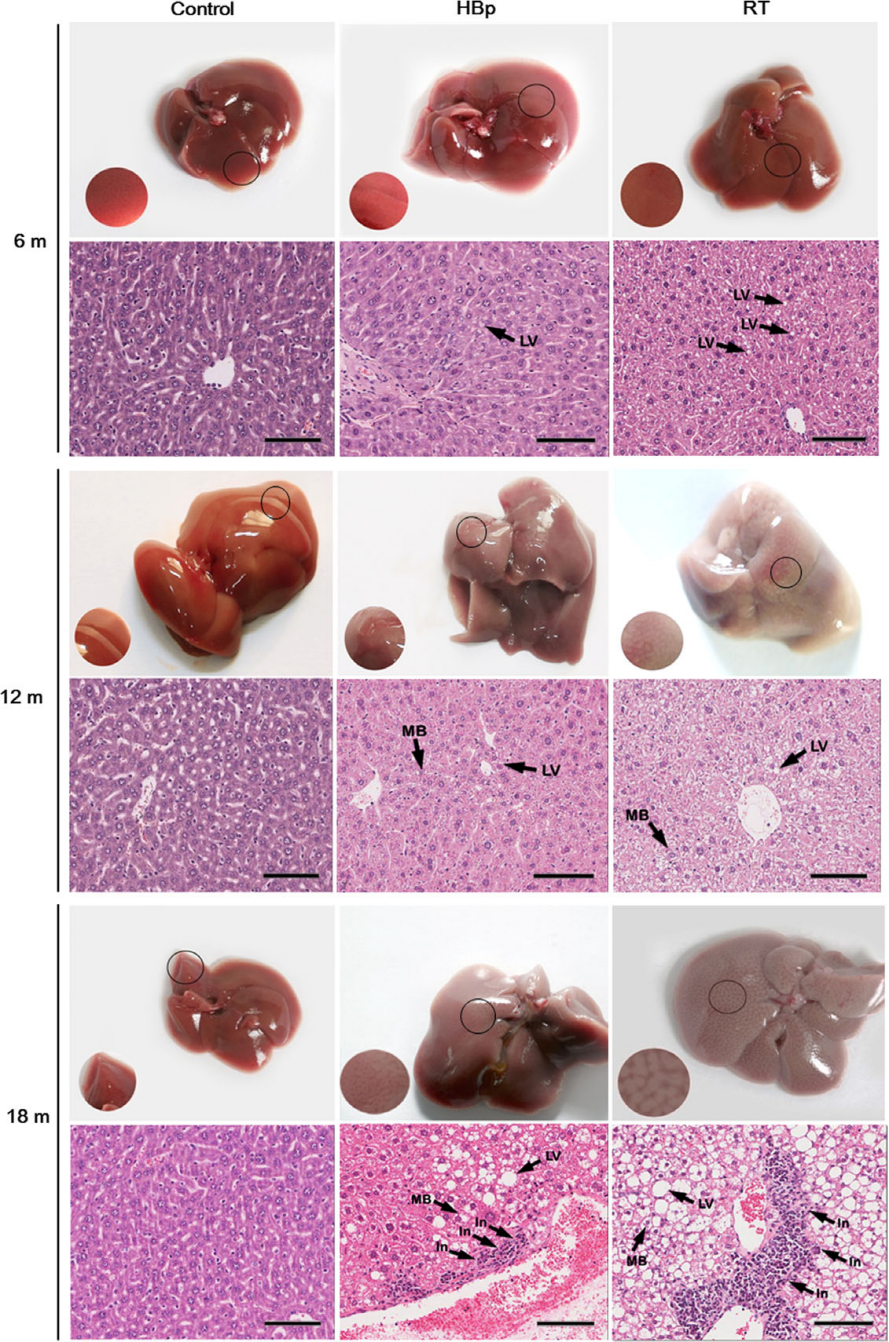
Liver damage in HBp and RT mice observed by histological examination. The morphological observations and histological examinations after H&E staining transgenic liver sections from 6‐, 12‐, and 18‐month‐old mice revealed fatty liver characteristics, LV, MB, and inflammation (In), as labeled in the image. For each group, representative images with hallmarks of liver diseases are shown (*n* = 10 mice per group). Scale bar, 100 μm.

The definitive approach for elucidating the etiological roles of genes in diseases is to observe transgenic mice expressing the genes. To investigate the possible role of HBp in association with the pathogenesis of CHB‐induced liver diseases, we generated transgenic mice expressing HBp specifically in the liver. The *HBp* gene (Fig. S1A) from the liver tissue of a CHB surface antigen (HBsAg) carrier 15, 16 was cloned downstream of the alpha‐1‐anti‐trypsin promoter for liver‐specific expression (GenBank accession No. D38257, Fig. S1B). The resulting *HBp* construct was microinjected into the male pronucleus of fertilized eggs. Six HBp transgenic mouse lines were identified, and the stable integration of the intact transgene into the host genome was confirmed by Southern blot analysis. All of these mice were bred out into permanent lines, thereby establishing HBp transgenic mice. A gross analysis of the livers of the HBp mice at 1 year of age showed obvious liver damage, indicating that HBp plays an important role in the pathogenesis of CHB‐induced liver diseases. Because the expression of *HBp* in the liver resulted in liver damage as expected, the 1.2‐kb *RT* domain of *HBp* was subcloned from the *HBp* gene to assess RT activity. After confirming that the RT domain had much stronger RT activity than HBp, the *RT* gene was cloned downstream of the alpha‐1‐anti‐trypsin promoter for liver‐specific expression (Fig. 1). The resulting *RT* construct was used to generate seven permanent lines of transgenic mice expressing *RT* in the liver.

If the expression level of a transgene is different from the expression level of another transgene, the effects of two genes on the pathogenesis of a disease cannot be compared directly because it is unclear whether the phenotypic differences in the two genes are rooted in functional‐ or expression‐level differences. As different expression levels of transgenes can make it difficult to evaluate the precise impact of the transgenes on chronic liver damage, we selected HBp and RT transgenic mouse lines with equivalent transgene expression levels by a gene expression analysis using real‐time PCR (Fig. S2). After selecting transgenic mice expressing equivalent levels of either *HBp* or *RT* transgenes in their livers, the integration sites of the transgenes in the HBp and RT mice were identified to eliminate the possibility of insertional mutagenesis that may lead to the misinterpretation of the effects of the transgenes. By alternative PCR, the transgene integration loci were identified as NT039492.8 (map position: 26.7 Mbp) on chromosome 10 for the RT mice and NT039472.8 (map position: 30.6Mbp) on chromosome 9 for the HBp mice. These integration loci excluded the possibility of insertional mutagenesis in the transgenic mice. The liver‐specific expression levels of the transgenes cloned downstream of the alpha‐1‐anti‐trypsin promoter were confirmed analyzing the total RNA extract of each mouse tissue sample by RT‐PCR (Fig. S3). As shown in Fig. S3, both transgenes were expressed specifically only in the liver during the experimental period, and the expression levels were stably maintained, showing typical gene expression levels of ordinary liver genes.

### The blood concentrations of biomarkers representing liver damage were increased in both HBp and RT mice, but were greater in RT mice

If the liver is damaged, liver proteins are released into the blood. Therefore, the blood concentrations of liver proteins are known to be increased in livers damaged by steatosis, cirrhosis, or HCC 18. As shown in Table 1, the serum concentrations of TIMP‐1, AST, ALT, collagen type IV, and hyaluronan were increased in an age‐dependent manner in both types of transgenic mice. Interestingly, the blood concentrations of the liver proteins were more severely elevated in RT mice than in HBp mice, meaning that the livers of the RT mice were more severely damaged than those of the HBp mice.

**Table 1:** Abnormal serum profiles indicating liver damage in HBp and RT mice. The data are presented as the mean ± SEM (*n* = 10 mice per group). *P* values ≤ 0.05 were considered significant

	Month 6	Month 12	Month 18
TMP‐1 (ng·mL^−1^)			
Control	1.1 ± 0.2	1.1 ± 0.1	1.1 ± 0.2
HBp	1.1 ± 0.1	2.1 ± 0.3^a^	2.8 ± 0.2^b^
RT	2.0 ± 0.1^c^	2.5 ± 0.2^c^	3.7 ± 0.2^c^
AST (ng·mL^−1^)			
Control	10.1 ± 0.5	10.1 ± 0.6	10.3 ± 0.5
HBp	11.4 ± 0.5	13.5 ± 1.7	15.8 ± 0.2^b^
RT	18.8 ± 1.6^a^	22.3 ± 1.9^a^	26.0 ± 0.4^c^
ALT (ng·mL^−1^)			
Control	14.4 ± 0.6	14.3 ± 0.5	14.2 ± 0.4
HBp	15.3 ± 0.3	16.8 ± 0.8^a^	19.6 ± 2.1^b^
RT	15.5 ± 0.3^a^	20.0 ± 0.7^b^	23.7 ± 0.9^c^
AST/ALT ratio			
Control	0.7 ± 0.01	0.8 ± 0.01	0.7 ± 0.02
HBp	0.8 ± 0.1	0.8 ± 0.1	0.8 ± 0.1
RT	1.2 ± 0.1^a^	1.2 ± 0.1^a^	1.1 ± 0.1^b^
Collagen IV (ng·mL^−1^)			
Control	83.4 ± 0.8	83.7 ± 1.0	84.0 ± 2.9
HBp	82.3 ± 0.7	91.5 ± 7.2	100.7 ± 1.9^a^
RT	90.0 ± 0.6	117.1 ± 9.5^a^	165.1 ± 7.5^c^
Hyaluronan (nmol·L^−1^)			
Control	24.9 ± 2.0	25.4 ± 2.4	25.5 ± 2.3
HBp	23.5 ± 1.5	35.2 ± 3.2	39.9 ± 3.2^a^
RT	25.8 ± 2.0	38.7 ± 2.6^a^	43.6 ± 3.5^b^
Blood glucose (mg·dL^−1^)			
Control	226.2 ± 7.8	229.8 ± 4.2	229.4 ± 3.8
HBp	219.0 ± 5.3	193.4 ± 7.0^b^	175.2 ± 5.0^b^
RT	212.0 ± 3.7	176.8 ± 6.6^c^	108.2 ± 6.4^c^
ALB (mg·mL^−1^)			
Control	29.2 ± 3.8	29.3 ± 4.3	28.9 ± 3.7
HBp	23.9 ± 2.9	19.4 ± 3.1^a^	13.6 ± 2.4^b^
RT	17.2 ± 2.2^b^	11.5 ± 2.0^c^	9.2 ± 2.2^c^
TC (mg·dL^−1^)			
Control	97.6 ± 8.0	97.3 ± 3.4	97.5 ± 3.0
HBp	97.3 ± 1.7	102.3 ± 7.6	105.6 ± 2.1
RT	103.1 ± 5.2	111.4 ± 6.6^a^	130.7 ± 1.3^c^
TG (mg·dL^−1^)			
Control	41.1 ± 9.7	46.2 ± 3.5	45.8 ± 4.0
HBp	51.8 ± 3.9	85.6 ± 16.4^a^	108.9 ± 9.2^b^
RT	48.3 ± 4.8	98.8 ± 21.1^a^	128.6 ± 25.1^c^
HDL cholesterol (mg·dL^−1^)			
Control	74.8 ± 6.9	74.8 ± 6.9	74.8 ± 6.9
HBp	73.0 ± 5.7	72.3 ± 10.6	70.5 ± 6.1
RT	72.1 ± 8.6	69.2 ± 7.6	68.6 ± 10.7
LDL cholesterol (mg·dL^−1^)			
Control	14.5 ± 4.7	12.7 ± 1.9	17.2 ± 1.3
HBp	13.2 ± 3.5	9.4 ± 4.6	9.9 ± 2.6
RT	13.5 ± 2.3	26.7 ± 6.3^a^	36.4 ± 4.0^b^
TGF‐β1 (ng·mL^−1^)			
Control	91.5 ± 5.7	91.1 ± 6.7	93.7 ± 5.3
HBp	97.5 ± 3.5	109.4 ± 8.6^b^	118.5 ± 3.1^c^
RT	101.5 ± 2.9	109.3 ± 3.8^b^	121.6 ± 3.9^c^
MCP‐1 (pg·mL^−1^)			
Control	18.6 ± 2.4	18.6 ± 2.9	19.7 ± 1.4
HBp	18.6 ± 1.7	21.2 ± 1.9	25.5 ± 2.5
RT	19.6 ± 1.8	22.3 ± 2.1	28.9 ± 3.4^a^
ICAM‐1 (ng·mL^−1^)			
Control	17.2 ± 1.1	17.3 ± 0.5	17.3 ± 1.6
HBp	15.6 ± 0.9	15.8 ± 1.5	17.7 ± 1.1
RT	16.3 ± 1.3	16.2 ± 1.3	18.2 ± 0.7

Values in a column with a superscripted letter are statistically significant as analyzed by a paired Student's *t*‐test; ^a^ *P* ≤ 0.05 compared with the control; ^b^ *P* ≤ 0.01 compared with the control; ^c^ *P* ≤ 0.001 compared with the control.

With elevated AST and ALT levels, the AST/ALT ratio is a clue to the etiology of underlying liver diseases. In conditions of elevated serum AST and/or ALT levels, the AST/ALT ratio is usually > 2.0 in alcoholic liver disease and < 1.0 in patients with chronic hepatitis or chronic cholestatic syndromes 19. However, the AST/ALT ratio range from 1.0 to 2.0 in HBV‐induced cirrhosis 20. Although the AST/ALT ratio of HBp mice was < 1.0, the AST/ALT ratio of RT mice was > 1.0 (Table 1). The AST/ALT ratio observed in RT mice, ranging from 1.1 to 1.2 (Table 1), corresponded to values typically observed in human CHB‐induced cirrhosis patients. Based on the AST/ALT ratio, RT mice developed cirrhosis starting from 6 months of age, while the status of the HBp mice was unclear. However, considering the overall liver function test (LFT) results of the HBp and RT mice, it should be noted that liver malfunction started to develop as early as 6 months of age in RT mice and 1 year of age in HBp mice.

### Hyperlipidemia and decreased glucose and albumin (ALB) blood concentrations were observed in HBp and RT mice, with greater abnormalities in RT mice

Because the liver is a vital organ for maintaining the dynamic balance of cholesterol and TGs, liver abnormalities caused by cirrhosis or CHB often lead to disturbances in the metabolism of cholesterol and TGs, resulting in abnormal cholesterol and TG concentrations 21, 22. Body weight gain and increased blood lipid profiles are positively associated with cirrhosis 21, 22, 23. As shown in Table 1, the blood lipid levels in these transgenic mice were also elevated in an age‐dependent manner, with greater changes in observed in RT mice. In agreement with the hyperlipidemia and liver steatosis of the transgenic mice, the transgenic mice were overweight compared with the littermate controls under a normal low‐fat diet (Fig. S4). While the HBp mice weighed slightly more than the controls, the RT mice were significantly more obese than the controls (Fig. S5).

Liver function is lost as cirrhosis progresses, thereby lowering the blood levels of ALB and glucose. These phenomena were also observed in the transgenic mice. Again, the symptoms were more severe in RT mice than in HBp mice (Table 1). Altered lipid and glucose blood profiles, such as hyperlipidemia and hypoglycemia resulting from liver failure, are typical complications of cirrhosis 24. Both types of transgenic mice again exhibited symptoms similar to those of human CHB‐induced cirrhosis patients.

### The RT domain of HBp stimulated proinflammatory cytokine secretion

Next, we conducted blood tests to assess serum inflammatory marker levels in the transgenic mice. As shown in Table 1, the inflammatory markers 25 TGF‐β1 and MCP‐1 were slightly elevated in the blood of both transgenic mice types, while ICAM‐1 was not elevated. Interestingly, the serum levels of TGF‐β1 and MCP‐1 were more elevated in RT mice than in HBp mice, as with the other serum pathological hallmarks shown in Table 1. These results suggest that the RT domain of HBp stimulated the secretion of proinflammatory cytokines to cause a low degree of inflammation and that the low‐grade inflammation observed in CHB might be rooted in RT activity.

### Histological examinations showed liver abnormalities in both HBp and RT mice, with greater liver abnormalities in RT mice

In H&E staining on the livers of transgenic mice, one of 10 HBp mice and two of 10 RT mice showed abnormal lipid vacuoles (LV) at 6 months of age, unlike the littermate controls (Fig. 1). The morphological shape of the LV clearly indicated steatosis in the histological liver sections. As the transgenic mice aged, the prevalence of steatosis increased dramatically. Seven of 10 HBp mice and nine of 10 RT mice had developed steatosis at 12 months of age. In addition, all 10 RT and 10 HBp mice had developed steatosis at 18 months of age (Table S2). In addition to the increased prevalence of steatosis, the pathological features in the transgenic mice became more clearly manifested as the mice aged. Extensive ballooning degeneration, Mallory body (MB) formation, and the formation of many LV were widely observed in the hepatocytes of 12‐month‐old transgenic mice (Fig. 1). These morbid pathological features of the hepatocytes were further intensified in the 18‐month‐old mice (Fig. 1). Additionally, the H&E staining results of the older transgenic mice were similar to those of cirrhosis with steatosis, suggesting that the transgenic mice could have cirrhosis with steatosis. Overall, the degrees of the pathological signs as well as the incidence rate of cirrhosis were more serious in RT mice than in HBp mice (Fig. 1). These histological examinations were consistent with the blood profile analysis results of the transgenic mice, in which RT mice exhibited more serious symptoms than HBp mice.

Other than more serious pathological signs and a higher incidence rate of cirrhosis in the RT mice, one of 10 RT mice suffering from cirrhosis with steatosis also developed HCC (Fig. S6 and Table S2). In CHB, cirrhosis is much more prevalent than HCC, and HCC does not develop alone 26, 27. HCC develops in patients with cirrhosis in most CHB cases 26, 27. This observation of the development of HCC in the background of cirrhosis suggests that the RT domain of HBp is an etiological agent of CHB‐induced liver diseases.

**Figure 2:**
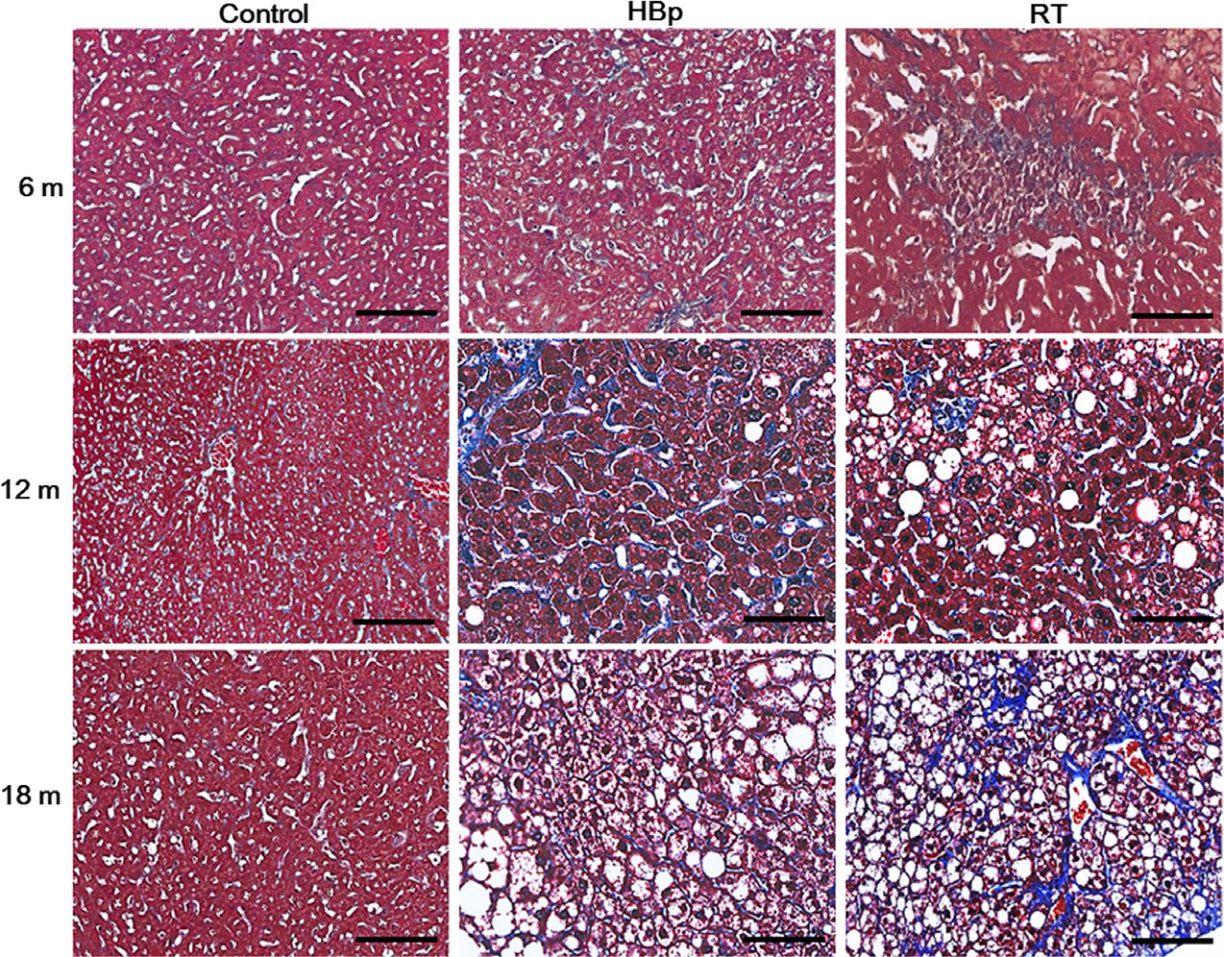
Liver damage in HBp and RT mice observed by histological examination after MT staining. After MT staining, the histological examination of transgenic liver sections from 6‐, 12‐, and 18‐month‐old mice revealed an early cirrhosis phenotype. For each group, representative images with hallmarks of liver diseases are shown (*n* = 10 mice per group). Scale bars, 100 μm.

The liver sections of the transgenic mice were further stained with MT. As shown in Figs 1 and 2, the liver sections showing steatosis by H&E staining (Fig. 1) also showed slight MT staining (Fig. 2), indicating the presence of fibrogenesis in the livers with steatosis. Based on the H&E and MT staining results, it was obvious that the 6‐month‐old diseased transgenic mice, one of 10 HBp mice, and two of 10 RT mice suffered from early cirrhosis with steatosis. As the transgenic mice aged, the prevalence of early cirrhosis increased dramatically. Seven of 10 HBp mice and nine of 10 RT mice developed early cirrhosis with steatosis at 12 months of age. In addition, all 10 RT and 10 HBp mice developed early cirrhosis with steatosis at 18 months of age (Table S2).

**Figure 3:**
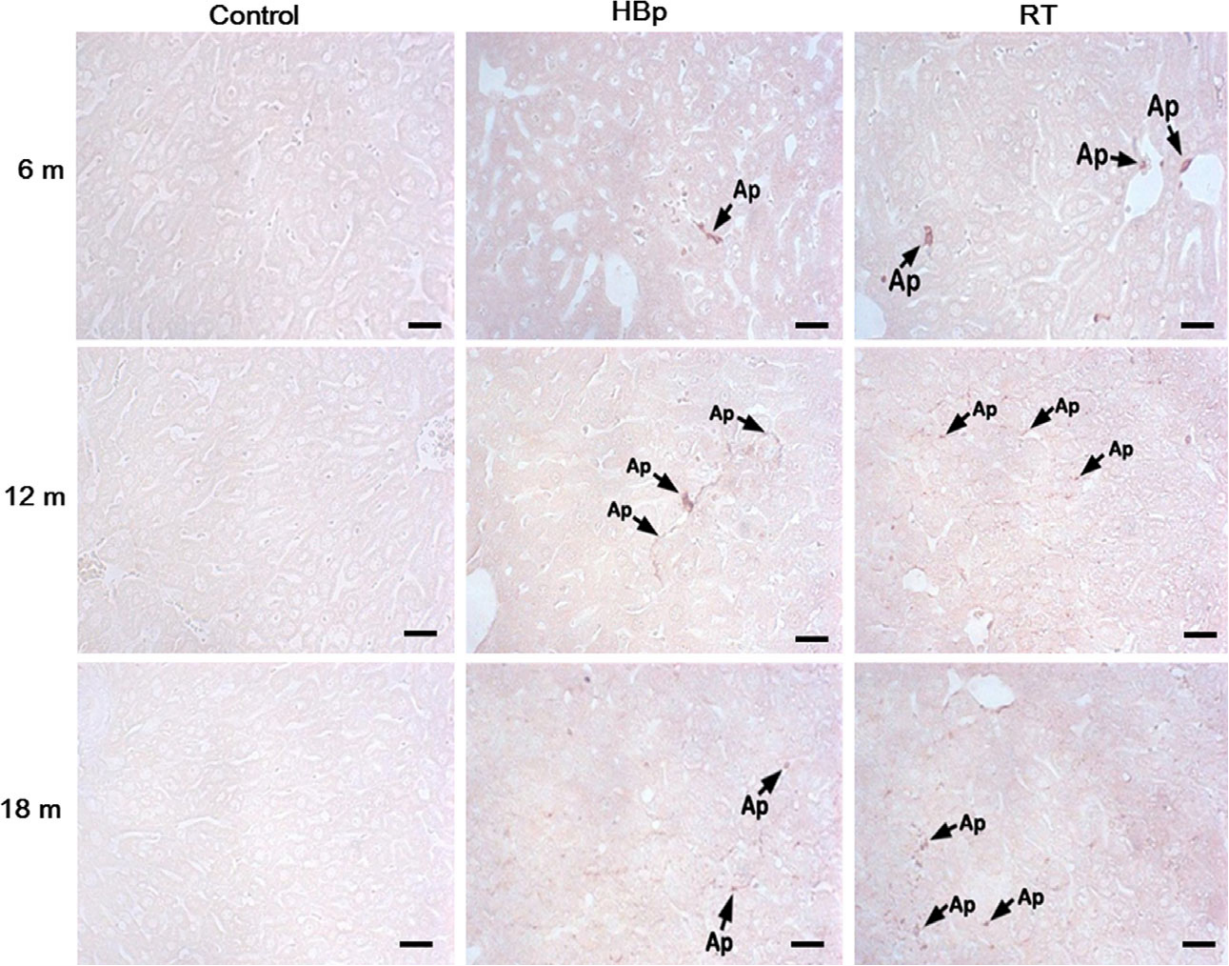
Liver damage in HBp and RT mice observed by histological examination after TUNEL staining. A histological examination after TUNEL staining transgenic liver sections from 6‐, 12‐, and 18‐month‐old mice revealed apoptosis characteristics (arrow, Ap). For each group, representative images with hallmarks of liver diseases are shown (*n* = 10 mice per group). Scale bar, 100 μm.

The histological examinations showed typical apoptotic features in the morbid hepatocytes, such as the formation of apoptotic bodies, cytoplasmic membrane blebbing, and a lack of nuclei (Fig. 3). TUNEL staining of the livers of transgenic mice confirmed that the morbid hepatocytes underwent major apoptosis, while apoptotic hepatocytes were not observed in the littermate controls (Fig. 3). Although there were no significant alterations in the shape of hepatocytes or the appearance of the liver in the transgenic mice with early signs of cirrhosis at 6 months (Fig. 1), slightly poor cytoplasmic staining and apoptotic cells were already beginning to be observed in the liver sections (Fig. 3). Taken together, as shown in Table 1 and Figs 1, 2, 3, the results of both the LFT and histological examinations showed that the degrees of the pathological signs were much more serious in RT mice than in HBp mice. There was also a higher incidence rate of early cirrhosis in RT mice than in HBp mice.

### The RT activity of HBp develops CHB‐induced liver diseases, such as cirrhosis, HCC, and steatosis, through coordinated proapoptotic and proinflammatory responses involving the caspase‐9, caspase‐3, and caspase‐1 pathways

**Figure 4:**
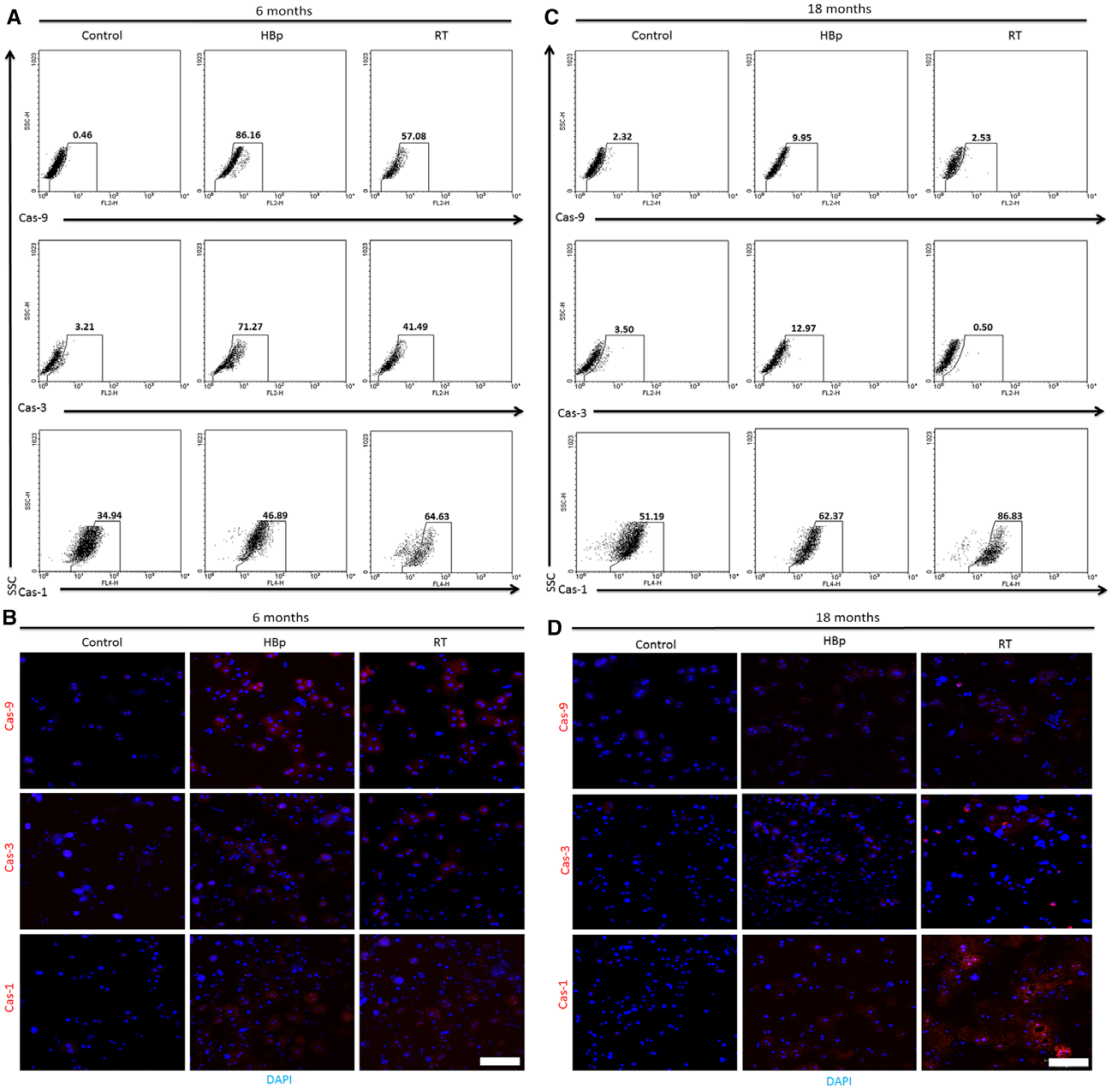
Activation of the caspase‐9‐ and caspase‐3‐dependent intrinsic apoptotic pathway and the caspase‐1‐dependent inflammatory pathway in the livers of HBp and RT mice. (A) Flow cytometry analyses of hepatocytes stained for caspase‐9, caspase‐3, and caspase‐1 after isolation from 6‐month‐old control, HBp, and RT mice. The numbers on the selected areas indicate the percentage of positive cells in those areas relative to the total number of hepatocytes. (B) Immunofluorescence microscopy images of anti‐caspase‐9, anti‐caspase‐3, and anti‐caspase‐1 in the same hepatocytes analyzed by flow cytometry shown in A. (C) Flow cytometry analyses of hepatocytes stained for caspase‐9, caspase‐3, and caspase‐1 after isolation from 18‐month‐old control, HBp, and RT mice. The numbers on the selected areas indicate the percentage of positive cells in those areas relative to the total number of hepatocytes. (D) Immunofluorescence microscopy images of anti‐caspase‐9, anti‐caspase‐3, and anti‐caspase‐1 in the same hepatocytes analyzed by flow cytometry shown in (C). Scale bar, 100 μm.

As the histological examinations indicated that the hepatocytes of the transgenic mice were dying through apoptosis to cause early cirrhosis with steatosis, apoptotic pathways were investigated by flow cytometry and confocal microscopy. Caspase‐9 and caspase‐3 were highly activated in the hepatocytes of young transgenic mice (Fig. 4), indicating that RT and HBp induce apoptosis through the caspase‐9‐ and caspase‐3‐dependent intrinsic apoptotic pathway. In this apoptotic pathway, activated caspase‐3 translocates from the cytoplasm into the nucleus after the induction of apoptosis. Thus, most of the active caspase‐3 is observed in the nucleus 28, 29. The flow cytometry and confocal microscopy data of 18‐month‐old mice showed typical examples of activated caspase‐3 localized in the nucleus, indicating end‐stage apoptosis in 18‐month‐old transgenic mice. The flow cytometry and confocal experiments also showed that the caspase‐1‐dependent inflammatory pathway was activated in the hepatocytes of the transgenic mice. In accordance with the LFT and histological examinations (Table 1, Figs 1, 2, 3), the caspase‐1‐dependent inflammatory pathway was more highly activated in RT mice than in HBp mice, and the inflammatory pathway became more activated as the transgenic mice aged.

### The RT activity of HBp is an etiological agent of CHB‐induced liver diseases, such as cirrhosis, HCC, and steatosis

**Figure 5:**
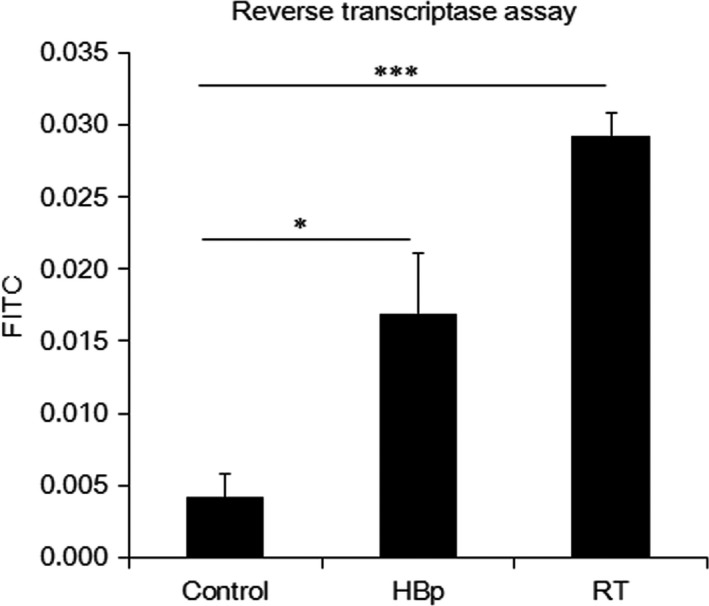
Correlation of RT activity and liver damage. RT activity was assessed by the cDNA synthesis capability of the cell extracts from the primary hepatocytes of 6‐month‐old control and transgenic mice. The fluorescence signal intensities of the littermate control, HBp, and RT mouse samples are proportional to RT activities. The data are presented as the mean ± SEM (*n* = 5 mice per group) and were compared by paired Student's *t*‐tests. *P* values ≤ 0.05 were considered significant (*P ≤ 0.05, ***P ≤ 0.001).

Recently, it was shown that incomplete reverse transcripts resulting from RT activity activate coordinated proapoptotic and proinflammatory responses, resulting in the death of HIV‐infected CD4 T cells 30. Because coordinated proapoptotic and proinflammatory activities in the livers of transgenic mice were evident by the blood profile analyses, histological examinations, and flow cytometry analyses (Table 1, Figs 1, 2, 3, 4), we examined whether the RT activity in the livers of the transgenic mice was etiologically responsible for the CHB‐induced liver diseases. As shown in Fig. 5, RT activity was much higher in RT mice than in HBp mice (Fig. 5). The fact that the prevalence and severity of the CHB‐induced liver diseases cirrhosis, steatosis, and HCC were positively correlated with RT activity indicates that the RT activity of the RT domain in HBp is an etiological agent of these CHB‐induced liver diseases.

## Discussion

In active CHB in humans, the following phenomena are commonly observed: Cirrhosis develops concurrently with steatosis, the incidence rate of cirrhosis is much more frequent than that of HCC, and HCC does not develop independently in most cases but rather develops in patients with cirrhosis 26, 27. These clinical consequences of human CHB are basically the same as the disease progression observed in our RT mice, in which cirrhosis developed much more frequently than HCC and HCC arose in cirrhotic livers. As shown in Table 1 and Figs 1, 2, 3, the degrees of the pathological signs of liver diseases in RT mice were much more serious than in HBp mice, which means that the RT domain of HBp is the etiological agent in CHB.

It should be noted that the viral protein production in CHB is limited so that the concentration of viral proteins in CHB is not as much as in the RT mice. All of the RT mice developed cirrhosis by mid‐age of the mice. However, only 20–30% of CHB develops liver diseases such as cirrhosis during their lifetime. We think this difference is rooted in the expression difference in the viral proteins between the RT mice and CHB. The disease progression of the RT mice not only matched the clinical consequences of CHB, but the disease phenotypes of the RT mice were also basically identical to those of CHB‐induced cirrhosis patients. The degree of the blood profile abnormalities in the RT mice (Table 1) was similar to that of the blood profile abnormalities in CHB‐induced cirrhosis patients. The histological examination results (Figs 1, 2, 3) confirmed that all the RT mice developed cirrhosis by 18 months of age. The pathological appearances of the cirrhotic livers in the RT mice revealed by the histological examinations were almost identical to those of CHB‐induced cirrhotic livers in humans. However, staining the morbid transgenic livers with MT showed that the RT mice suffered from early cirrhosis. Although liver fibrosis was widely observed in the morbid transgenic livers, the formation of cirrhotic nodules surrounded by fibrous scars was not observed in these transgenic mice. The regeneration of healthy undamaged hepatocytes around fibrous scars is known to form cirrhotic nodules 31. Considering that all hepatocytes in the transgenic mice have the same pathogenic gene, that is, either *HBp* or *RT*, it is not surprising that cirrhotic nodules were not observed in these transgenic mice. Additionally, considering that 18 months in mice is equivalent to middle age in human years, it would be reasonable to speculate that the cirrhotic livers of the RT mice would develop cirrhotic nodules in later age, as in cases of end‐stage cirrhosis in humans.

Our results suggest that the RT activity of HBp stimulates the activation of caspase‐9 and caspase‐3, which are linked to apoptosis, as well as caspase‐1, which promotes the processing and secretion of proinflammatory cytokines, such as TGF‐β1 and MCP‐1, which promote systemic inflammation 32, 33. These proinflammatory cytokines are known to be caspase‐1 dependent. Previous works have shown that the activation of caspase‐1 in hepatocytes stimulated them to secrete the proinflammatory cytokines TGF‐β1 and MCP‐1 34, 35. Our flow cytometry analyses (Fig. 4) also suggested that the elevated blood concentrations of TGF‐β1 and MCP‐1 (Table 1) could be consequences of caspase‐1 activation. Although the expression levels of TGF‐β1 and MCP‐1 in hepatocytes were moderately increased in the HBp and RT mice, the quantities of these proinflammatory cytokines were sufficient to cause constant, low‐level inflammation, which is a typically observed symptom in human CHB.

Recently, Doitsh et al. 30 showed that the RT activity in HIV‐infected CD4 T cells is responsible for their cell death by activating a host defense program that elicits coordinated proapoptotic and proinflammatory responses involving caspase‐3 and caspase‐1 activation. The experimental results showing that RT leads to the death of HIV‐infected CD4 T cells are basically the same as our experimental results, in which the RT domain of HBp causes the death of hepatocytes, leading to cirrhosis and HCC. As in the case of HIV‐infected CD4 T cells, the RT activity of HBp activated coordinated proapoptotic and proinflammatory responses involving caspase‐9, caspase‐3, and caspase‐1 activation (Figs 4 and 5).

**Figure 6:**
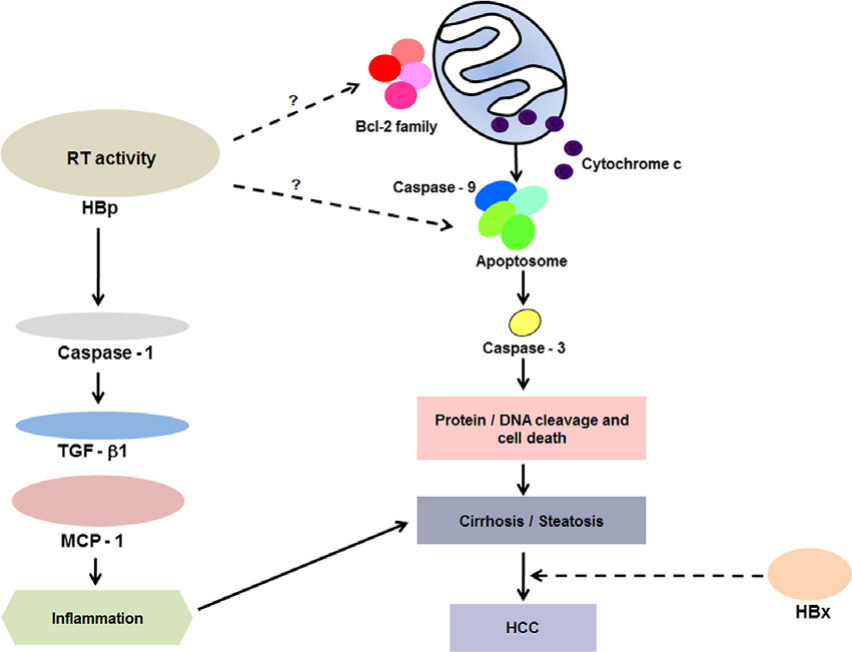
The signaling pathways inducing liver diseases in CHB. The coordinated proapoptotic and proinflammatory responses involving the caspase‐9/caspase‐3 and caspase‐1 pathways are activated by the RT activity of HBp. HBx might play an amplifying role in RT activity‐induced liver diseases.

There are two well‐known apoptotic signaling pathways in cells, the intrinsic and extrinsic pathways 36. While apoptosis‐inducing ligands are initiators of apoptosis in the extrinsic apoptotic pathway, in the other pathway, intrinsic signals mediated by mitochondria activate the initiator caspase, caspase‐9, which then activates the effector caspase, caspase‐3, which then cleaves many important cellular substrates and causes cell death. We showed that the RT activity of HBp activated both caspase‐9 and caspase‐3 (Fig. 4), meaning that the RT activity of HBp triggers the caspase‐3‐dependent intrinsic apoptotic pathway, eventually leading to the death of hepatocytes 37. Because the death of hepatocytes typically results in cirrhosis 38, the activation of the intrinsic apoptotic pathway via RT activity could act as a direct cause of hepatocyte death and the development of cirrhosis and HCC (Fig. 6). Other than the activation of caspase‐9 and caspase‐3, the RT activity of HBp also activated caspase‐1, which triggers the secretion of the proinflammatory cytokines TGF‐β1 and MCP‐1 from hepatocytes (Table 1, Fig. 4). Because increased levels of these proinflammatory cytokines secreted from the hepatocytes of transgenic mice could potentially induce low‐level inflammation, it would be reasonable to speculate that the activation of caspase‐1 via HBp RT activity plays an important role in the low degree of persistent inflammation in CHB that intensifies hepatocyte damage (Fig. 6). HBp RT activity alone could act as an etiological agent of CHB‐induced liver diseases. However, in addition to inflammation, HBx can also magnify HBx‐induced liver diseases because HBx has an oncogenic property and functions as a transactivator that can induce dysfunctional signaling pathway regulation, transcription, and cell cycle progression through interactions with target host factors (Fig. 6).

In summary, the current study demonstrates that the phenotypes of the RT mice were basically identical to the typical clinical consequences of CHB. In this context, it would be reasonable to speculate that HBp RT activity is an etiological agent of CHB‐induced liver diseases, such as cirrhosis, steatosis, and HCC, rather than HBx, which induces HCC under defined conditions 10, 39. The blood levels of HBsAg have been shown to be positively correlated with the incidence rate of CHB‐induced liver diseases 40. Because the expression levels and activities of HBV genes, including HBp, are best represented by the blood levels of HBsAg 40, the positive relationship between the HBsAg blood level and the incidence rate of CHB‐induced liver diseases also supports our finding that HBp RT activity plays a central etiological role in CHB‐induced liver diseases. Finally, these results indicate that our transgenic mice could serve as a good animal model for elucidating molecular events in CHB‐induced liver diseases and for showing the pathogenic potential of the RT embedded in every retrovirus and pararetrovirus.

## Author contributions

HJC, XC, CHS, HC, SL, and STH designed research. HJC, XC, YY, HKL, HJK, and STH performed research and analyzed data. HJC, XC, HJK, and STH wrote the manuscript with input of all authors.

## Supporting information


**Fig. S1.** Recombinant plasmids constructed to generate transgenic mice.
**Fig. S2.** Transgene expression levels in the liver.
**Fig. S3.** Comparative analyses of transgene expression in the transgenic mice at 6, 12, and 18 months after birth.
**Fig. S4.** Innate pro‐obesity nature of the transgenic mice in contrast to littermate controls.
**Fig. S5.** Body weight indicating liver damage in HBp and RT mice.
**Fig. S6.** HCC observed in an 18‐month‐old RT mouse.
**Table S1.** List of primers used in amplification.
**Table S2.** The abnormal number of transgenic mice diagnosed by histological examination.
