# Supplementary material for: A critical role of hepatitis B virus polymerase in cirrhosis, hepatocellular carcinoma, and steatosis
Source: FEBS Open Bio. 2017 Dec 19;8(1):130–45. doi: 10.1002/2211-5463.12357 (PMC5757181; doi:10.1002/2211-5463.12357)
Supplement: Supplementary file 1 — Fig. S1. Recombinant plasmids constructed to generate transgenic mice. Fig. S2. Transgene expression levels in the liver. Fig. S3. Comparative analyses of transgene expression in the transgenic mice at 6, 12, and 18 months after birth. Fig. S4. Innate pro‐obesity nature of the transgenic mice in contrast to littermate controls. Fig. S5. Body weight indicating liver damage in HBp and RT mice. Fig. S6. HCC observed in an 18‐month‐old RT mouse. Table S1. List of primers used in amplification. Table S2. The abnormal number of transgenic mice diagnosed by histological examination. [file FEB4-8-130-s001.docx]

**Supporting Information**

**A critical role of hepatitis B virus polymerase in cirrhosis, hepatocellular carcinoma, and steatosis**

Hea-Jong Chung, Xiao Chen, Yang Yu, Heui-Kwan Lee, Chang Ho Song, Han Choe, Seungkoo Lee, Hyeon-Jin Kim, and Seong-Tshool Hong


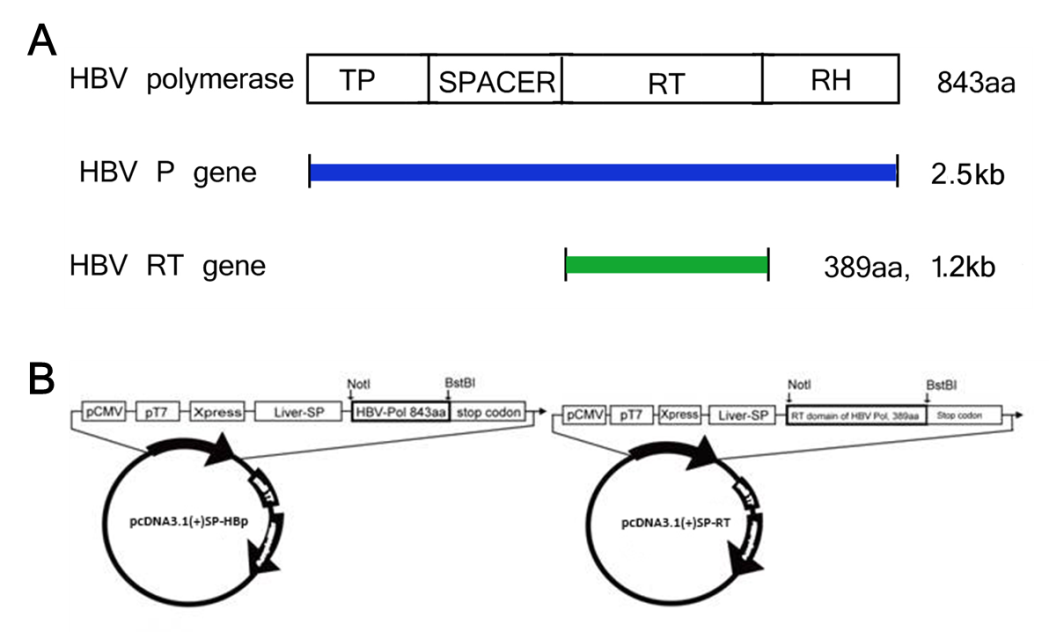


**Fig. S1.** Recombinant plasmids constructed to generate transgenic mice. (A) The DNA segments of *HBp* and *RT* genes used for plasmid construction. Terminal portion (TP), reverse transcriptase (RT), and RNase H (RH). (B) The recombinant plasmids containing the liver-specific promoter and target genes.


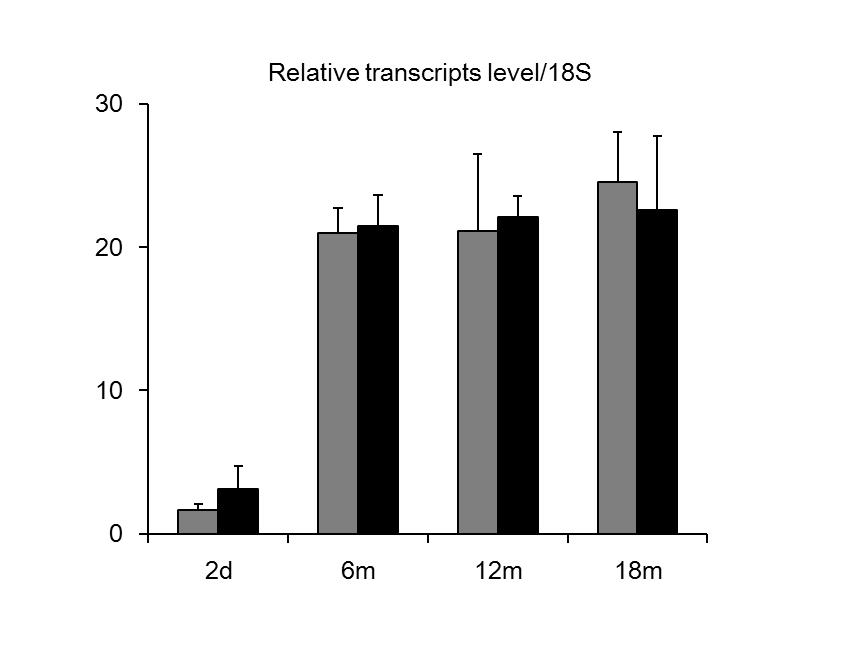


**Fig. S2.** Transgene expression levels in the liver. Transgene expression levels were determined using quantitative real-time PCR at 2 days and 6, 12 and 18 months after birth. Transcript levels were relative to 18S rRNA. Control groups are shown as white bars (□), HBp as grey bars (■), and RT as black bars (■). The data were presented as the mean ± s.e.m. (n = 10 mice per group).

**
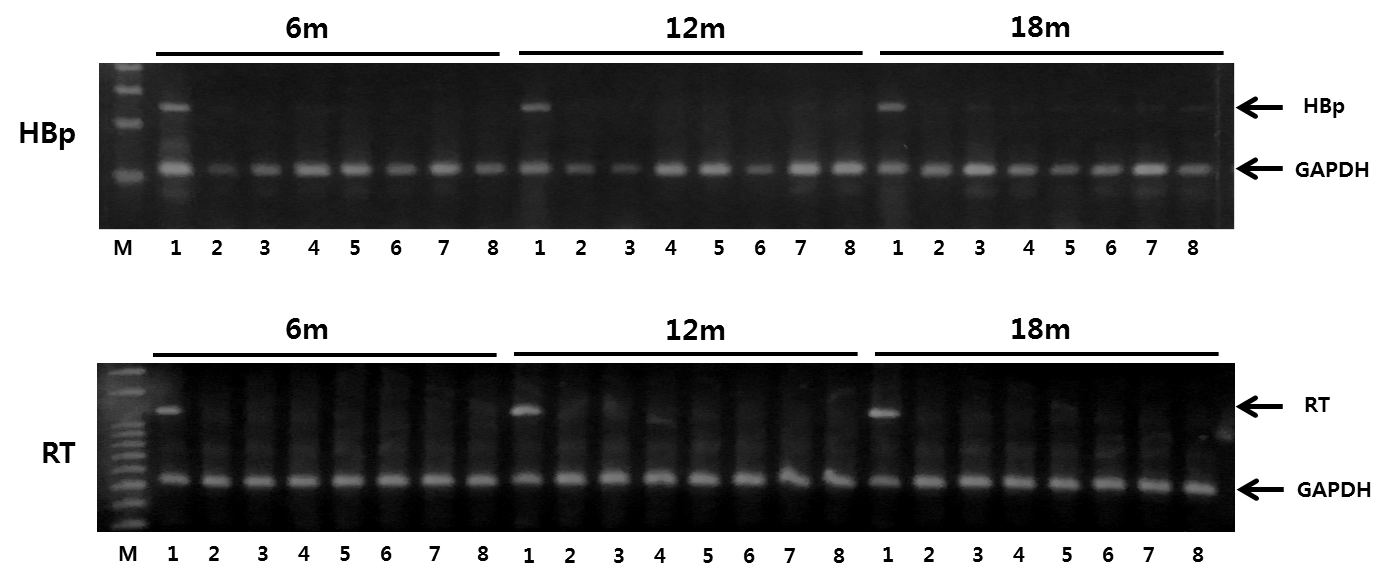
**

**Fig. S3.** Comparative analyses of transgene expression in the transgenic mice at 6, 12, and 18 months after birth. Total RNAs were isolated from the each organ of HBp or RT mice at 6, 12, and 18 months after birth to analyze gene expression of the transgenes, RT or HBp, by RT-PCR. M, Marker; Lane 1, Liver; 2, Lung; 3, Heart; 4, Muscle; 5, Kidney; 6, Stomach; 7, Spleen; 8, Brain

**
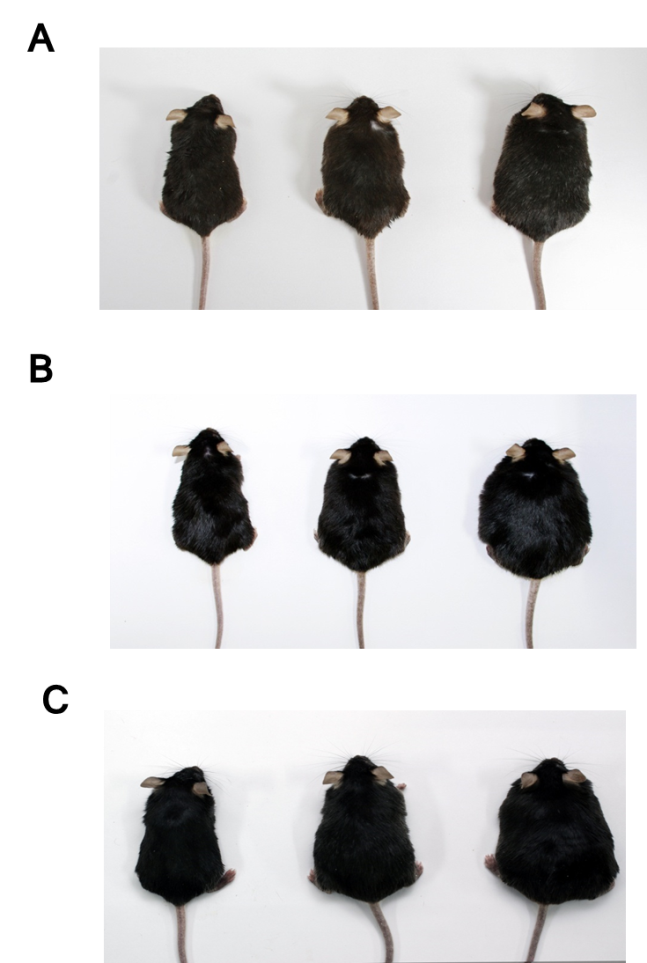
**

**Fig. S4.** Innate pro-obesity nature of the transgenic mice in contrast to littermate controls. The obesity phenotype of transgenic RT (right), HBp (middle) and littermate control mice (left) at 6 (A), 12 (B) and 18 months old (C).


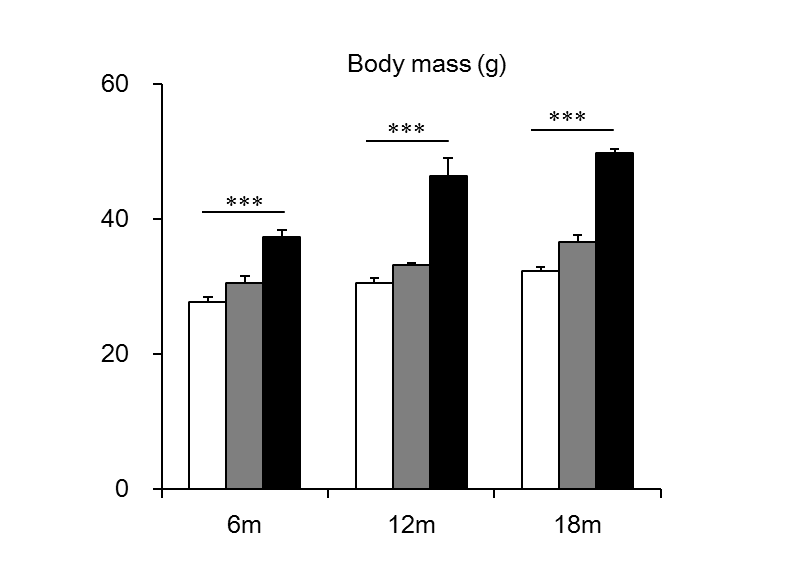


**Fig. S5.** Body weight indicating liver damage in HBp and RT mice. The body weights were performed on mice at 6, 12 and 18 months old. Control groups are shown as white bars (□), HBp as grey bars (■), and RT as black bars (■). The data were presented as the mean ± S.E.M. (n = 10 mice per group) and analyzed by a paired Student’s *t*-test for comparisons. *P* values ≤ 0.05 were considered significant (**P* ≤ 0.05, ***P* ≤ 0.01, ****P* ≤ 0.001).


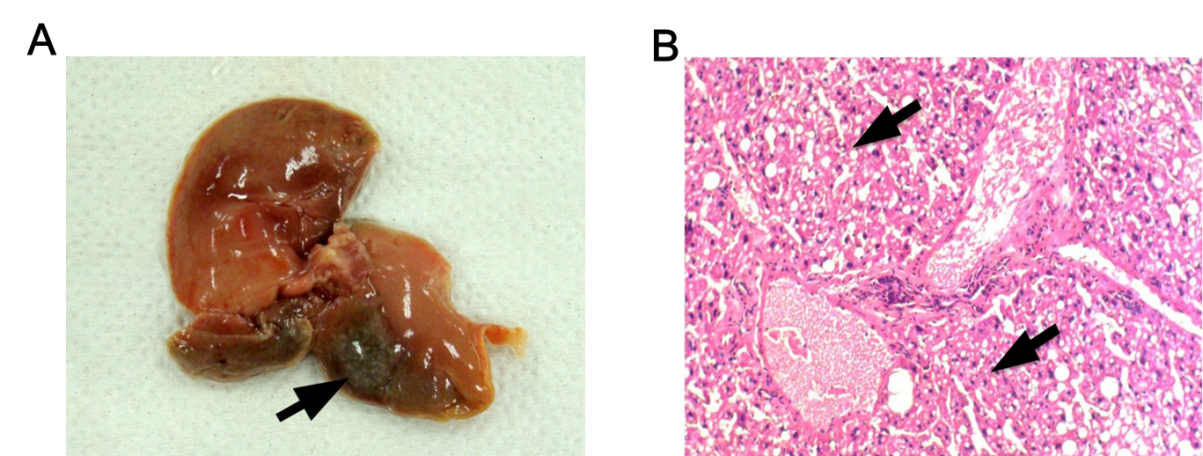


**Fig. S6.** Hepatocellular carcinoma observed in an 18-month-old RT mouse. (A) One of 10 RT mice showed a large mass lesion (arrow) in its liver. (B) The H & E staining on the liver section showed a typical characteristic of HCC in the mass lesion including thickness of cell cords with cellular atypia (arrow). Scale bars, 200 μm.

**Table S1.** List of primers used in amplification

|  | **Primer** | **Nucleotide Sequence (5'→ 3')** | **Approach** |
| --- | --- | --- | --- |
| Cloning | HBp-F | 5’-GTTGCGGCCGCATAATGGCCCTATCTTATC-3’ | PCR |
|  | HBp-R | 5’-ATTTTCGAATTCTCACGGTGGTTTCCA-3’ |  |
|  | RT-F | 5’-GTTGCGGCCGCTTAATGGACTACTGCCTCACC-3’ | PCR |
|  | RT-R | 5’-GAATTCGAAAATTCCTGACCGTTGCCGGGC-3’ |  |
|  | AAP-F | 5’-GCGAAGCTTTCTGAATGTGTGTGC-3’ | PCR |
|  | AAP-R | 5’-TATAATAGCGGCCGCGTGGTGGTG-3’ |  |
| Genotyping | HBp1-F | 5’-CTTTGCTGCCCCTTTTACACA-3’ | PCR |
|  | HBp1-R | 5’-GATGCCCCGATTTAGAGCTTGA-3’ |  |
|  | RT1-F | 5’-GGAGGTCTATATAAGCAGAGCTCT-3’ | PCR |
|  | RT1-R | 5’-AGTCATTGTACCTGGCTCAGAAA-3’ |  |
| Gene Expression | HBp2-F | 5’-TGGGCCTCAGTCCGTTTC-3’ | Real-time PCR |
|  | HBp2-R | 5’-GCCCTGCGAACCACTGAA-3’ |  |
|  | RT2-F | 5’-GCCAGCCTGACGGTTGTG-3’ | Real-time PCR |
|  | RT2-R | 5’-TCTTGATGGTCCCGATGGA-3’ |  |
|  | 18S rRNA-F | 5’-AGGGGAGAGCGGGTAAGAGA-3’ | Real-time PCR |
|  | 18S rRNA-R | 5’-GGACAGGACTAGGCGGAACA-3’ |  |
| Genomic localization | SP1-R | 5’-TCAGCAACCAGGTGTGGAAAGT-3’ | Alternative PCR |
|  | SP2-R | 5’-TGCTCTGATGCCGCCGTGTT-3’ |  |
|  | SP3-F | 5’-ATAGAAGGCGGCGGTGGAATC-3’ | Alternative PCR |
|  | SP4-F | 5’-CACGGGTAGCCAACGCTATGTC-3’ |  |
|  | AD1-r | 5’-NGTCGASWGANAWGAA-3’ | Alternative PCR |
|  | AD2-r | 5’-GTNCGASWCANAWGTT-3’ |  |
|  | AD3-r | 5’-WGTGNAGWANCANAGA-3’ |  |
|  | Restriction enzyme recognition sequences are underlined. F, forward; R, reverse; r, random.  HBp, hepatitis B viruse polymerase; RT, reverse transcriptase; AAP, alpha-1-antitrypsin gene promoter; SP, specific primer; AD, arbitrary degenerate. | | |

**Table S2.** The abnormal number of transgenic mice diagnosed by histological examination

|  | Littermate | | | HBp | | | RT | | |
| --- | --- | --- | --- | --- | --- | --- | --- | --- | --- |
|  | 6 m | 12 m | 18 m | 6 m | 12 m | 18 m | 6 m | 12 m | 18 m |
| Total number of mice | 10 | 10 | 10 | 10 | 10 | 10 | 10 | 10 | 10 |
| Early cirrhosis with steatosis | 0 | 0 | 0 | 1 | 7 | 10 | 2 | 9 | 10 |
| Hepatocellular carcinoma  (with early cirrhosis) | 0 | 0 | 0 | 0 | 0 | 0 | 0 | 0 | 1 |
| Normal mice | 10 | 10 | 10 | 9 | 3 | 0 | 8 | 1 | 0 |
